# Therapeutic education program in adults with unipolar depression

**DOI:** 10.1192/j.eurpsy.2023.1780

**Published:** 2023-07-19

**Authors:** N. Halouani, N. Regaieg, M. Turki, S. Ellouze, J. Aloulou

**Affiliations:** 1 Psychiatry B Department, Hedi Chaker University Hospital, Sfax, Tunisia, Faculty of Medecine of Sfax, Tunisia, Sfax, Tunisia

## Abstract

**Introduction:**

Depression is one of the most common chronic illnesses. It requires long-term multidisciplinary care, combining pharmacological and non-pharmacological treatments. Hence the need for an educational approach to improve the quality of life of these patients.

**Objectives:**

Our objective is to create a personalized educational program for patients followed for depression allowing them to acquire the necessary skills to become autonomous in the management of their pathologies on a daily basis.

**Methods:**

The therapeutic education program is aimed at patients followed for depression and their families. Our team is multidisciplinary made up of a psychiatrist, a nurse and a dietitian. The educational tools are rich and varied, including computerized resources, written information, brochures and educational games.

**Results:**

The first step is the educational diagnosis which allows to identify the personalized needs of the patient. The caregiver-educator sets with the patient the objectives to be achieved throughout the course, thus defining the educational contract. Then the patient and his entourage can follow a personalized therapeutic patient education program. We offer a program consisting of 7 sessions at the rate of one session per one to two months (2 individual sessions and 5 group workshops). At the end of the program, evaluation and self-evaluation grids are completed.

**Conclusions:**

Therapeutic patient education provides knowledge through which patients with depression develop personal and interpersonal coping skills. This program will allow them to give an acceptable place to their disease so that they can evolve well with it.

**Disclosure of Interest:**

None Declared

